# Exploring Microorganisms Associated to Acute Febrile Illness and Severe Neurological Disorders of Unknown Origin: A Nanopore Metagenomics Approach

**DOI:** 10.3390/genes15070922

**Published:** 2024-07-15

**Authors:** Keldenn Melo Farias Moreno, Virgínia Antunes de Andrade, Felipe Campos de Melo Iani, Vagner Fonseca, Maurício Teixeira Lima, Emerson de Castro Barbosa, Luiz Marcelo Ribeiro Tomé, Natália Rocha Guimarães, Hegger Machado Fritsch, Talita Adelino, Tatiana Oliveira Fereguetti, Maíra Cardoso Aspahan, Tereza Gamarano Barros, Luiz Carlos Junior Alcantara, Marta Giovanetti

**Affiliations:** 1 Institute of Biological Sciences, Federal University of Minas Gerais, Belo Horizonte 31270-901, Brazil; keldennmfmoreno@gmail.com (K.M.F.M.); maurili15@hotmail.com (M.T.L.); hegger.fritsch@gmail.com (H.M.F.); 2 Eduardo de Menezes Hospital, Belo Horizonte 30622-020, Brazil; virginiaantunesdeandrade@gmail.com (V.A.d.A.); tatianiofereguetti@gmail.com (T.O.F.); maiaspahan@gmail.com (M.C.A.); teresa.barros@fhemig.mg.gov.br (T.G.B.); 3 Central Public Health Laboratory of the State of Minas Gerais, Ezequiel Dias Foundation, Belo Horizonte 30510-010, Brazil; felipe.iani@funed.mg.gov.br (F.C.d.M.I.); talitadelino@gmail.com (T.A.); 4 Department of Exact and Earth Sciences, University of the State of Bahia, Salvador 41150-000, Brazil; vagner.fonseca@gmail.com; 5 René Rachou Institute, Oswaldo Cruz Foundation, Belo Horizonte 30190-002, Brazil; emersoncb7@gmail.com (E.d.C.B.); luiz.tome@funed.mg.gov.br (L.M.R.T.); natyroguiman@yahoo.com.br (N.R.G.); luiz.alcantara@fiocruz.br (L.C.J.A.); 6 Morphogenesis and Antigenicity of HIV and Hepatitis Viruses, University of Tours, 37032 Tours, France; 7 Department of Sciences and Technologies for Sustainable Development and One Health, Università Campus Bio-Medico di Roma, 00128 Rome, Italy; 8 Oswaldo Cruz Foundation, Rio de Janeiro 21040-900, Brazil

**Keywords:** acute febrile illness, severe neurological disorders, nanopore sequencing, metagenomics

## Abstract

Acute febrile illness (AFI) and severe neurological disorders (SNDs) often present diagnostic challenges due to their potential origins from a wide range of infectious agents. Nanopore metagenomics is emerging as a powerful tool for identifying the microorganisms potentially responsible for these undiagnosed clinical cases. In this study, we aim to shed light on the etiological agents underlying AFI and SND cases that conventional diagnostic methods have not been able to fully elucidate. Our approach involved analyzing samples from fourteen hospitalized patients using a comprehensive nanopore metagenomic approach. This process included RNA extraction and enrichment using the SMART-9N protocol, followed by nanopore sequencing. Subsequent steps involved quality control, host DNA/cDNA removal, de novo genome assembly, and taxonomic classification. Our findings in AFI cases revealed a spectrum of disease-associated microbes, including *Escherichia coli*, *Streptococcus* sp., Human Immunodeficiency Virus 1 (Subtype B), and Human Pegivirus. Similarly, SND cases revealed the presence of pathogens such as *Escherichia coli*, *Clostridium* sp., and Dengue virus type 2 (Genotype-II lineage). This study employed a metagenomic analysis method, demonstrating its efficiency and adaptability in pathogen identification. Our investigation successfully identified pathogens likely associated with AFI and SNDs, underscoring the feasibility of retrieving near-complete genomes from RNA viruses. These findings offer promising prospects for advancing our understanding and control of infectious diseases, by facilitating detailed genomic analysis which is critical for developing targeted interventions and therapeutic strategies.

## 1. Introduction

Acute febrile illness (AFI) is a clinical syndrome characterized by fever (≥38.0 °C) and often accompanied by various non-specific symptoms such as headache, rash, and muscle and joint pains. This illness frequently necessitates hospitalization and extensive investigations to determine its cause, which is primarily due to infectious pathogens that vary epidemiologically around the world. For instance, malaria is the predominant cause of AFI in Africa, whereas in Latin America and Asia, dengue and leptospirosis are more common causes of this syndrome [1,2].

Despite advancements in medical diagnostics, a significant proportion of AFI cases remain unresolved. Conventional diagnostic approaches often yield inconclusive or negative results. Studies indicate that nearly 50% of AFI cases remain undiagnosed, a figure that may be higher due to the frequent misdiagnosis of AFI as arboviral disease in primary care settings, where no further investigations are conducted [3,4].

Severe neurological disorders (SNDs) encompass a range of conditions that impact the brain, spinal cord, and nerves. Symptoms of SNDs may include headaches, taste and smell dysfunction, muscle weakness, paralysis, a loss of sensation, poor coordination, confusion, seizures, and pain. SNDs are the second leading cause of death globally, with various causes including infectious bacteria, fungi, and particularly viruses [5,6].

Due to the non-specific symptoms and the wide range of associated infections, SNDs and AFI present significant diagnostic challenges. In many cases, supplementary methodologies are necessary to determine the etiological origins of these complex diseases. Among these methodologies, metagenomics has emerged as a revolutionary approach. It provides a comprehensive analysis of the microbial composition within biological samples. This technique facilitates the identification of potential emerging and re-emerging pathogens through nucleotide sequence analysis and generates valuable genomic data. These insights enhance our understanding of disease biology, host–pathogen interactions, and epidemiology [7].

The application of metagenomics in the detection of infectious agents, especially those elusive to conventional diagnostic methods, has seen a marked increase in recent years [8,9]. Metagenomic analysis leverages a variety of next-generation sequencing (NGS) technologies. Among these, the MinION sequencing platform is increasingly favored due to its capacity to rapidly generate substantial data volumes. Its long-read sequencing capabilities address the challenges associated with ambiguous repetitive regions, enhancing genomic contiguity. The portability and cost-effectiveness of this technology further bolster its adoption in genomic research [10]. As the epidemiological landscape of infectious diseases continues to shift, the relevance of sophisticated metagenomic methodologies becomes more pronounced. These technologies are crucial in elucidating the intricate interactions between pathogens and their hosts, aiming to mitigate the public health burden of diseases such as AFI and SND. They are instrumental in safeguarding public health in an era characterized by the frequent emergence and re-emergence of infectious diseases.

In light of these capabilities, this study sought to employ the nanopore sequencing platform to identify and characterize microorganisms linked to febrile or neurological disorders of unknown etiology. The investigation focused on a cohort of hospitalized patients with undiagnosed cases of AFI or SND, demonstrating the utility of metagenomics in resolving complex diagnostic challenges in clinical settings.

## 2. Materials and Methods

### 2.1. Clinical Sampling

**Figure 1:**
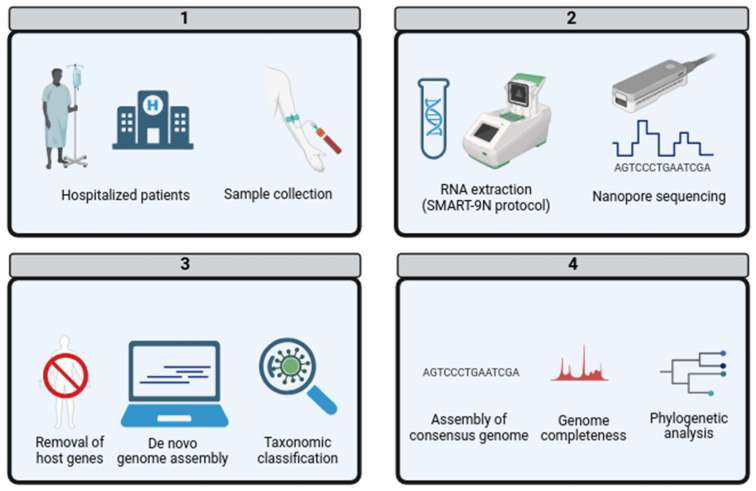
Metagenomic workflow analysis of AFI and SND in hospitalized patients. (**1**) Sample collection: Collection of biological samples from hospitalized patients diagnosed with acute febrile illness [AFI] or severe neurological disorder [SND] of unidentified origin. (**2**) Laboratory Processing: Samples underwent RNA extraction using the SMART-9N protocol, followed by metagenomic sequencing employing nanopore technology. (**3**) Data Analysis: Sequences were subjected to quality control, the removal of host genetic material, and the generation of contigs through de novo genome assembly. These contigs were then classified taxonomically. (**4**) Pathogen Identification and Further Analysis: Identified pathogens with coverage exceeding 80% were used to assemble a consensus genome. The completeness of these genomes was assessed, culminating in the construction of a phylogenetic tree.

The Eduardo de Menezes Hospital in Belo Horizonte, Brazil, plays a crucial role in healthcare by conducting etiological investigations for patients hospitalized with conditions such as acute febrile illness or severe neurological disorders. The hospital ensures the highest biosafety standards during the collection and identification of samples, following medical requests. Collaborating with the Ezequiel Dias Foundation (FUNED), the hospital aims to uncover possible links between infections with inconclusive diagnoses and microbial infections. This is carried out through extensive serological and molecular biology investigations targeting arboviruses, hepatitis, rickettsiosis, hantavirus, leptospirosis, and other pathogens. Samples are collected based on clinical information and medical requests and may include serum, whole blood, swabs, or cerebrospinal fluid. If an RNA virus is suspected, blood is also collected in a Tempus tube, which contains chemicals that preserve RNA for a longer period. Despite these efforts, in 2022, fourteen patients had inconclusive results even after serological and molecular biology investigations. As an alternative approach to identify the etiological agents, these patient samples were subjected to metagenomic analysis (Figure 1).

### 2.2. RNA Extraction and Nanopore Sequencing

RNA was extracted from a range of 200 to 500 μL of biological samples using the High Pure RNA Isolation Kit (Hoffmann–La Roche, Basel, Switzerland) and concentrated using the RNA Clean & Concentrator-5 (Zymo Research, Irvine, CA, USA). The samples then underwent the SMART-9N PCR protocol [11], utilizing a three-random-primed SMART-Seq model compatible with Oxford Nanopore Technologies (ONT) adapters. This protocol is particularly effective in enhancing the detection and sequencing of pathogen genomes, including RNA viruses, and facilitates the identification and sequencing of DNA viruses, bacteria, fungi, eukaryotes, and archaea. Library preparation involved multiple steps: PCR purification using AMPure XP beads (Beckman Coulter, Indianapolis, IN, USA), the normalization of DNA concentration, purification, end repair, and dA-tailing using the Ultra II End Prep Kit (New England Biolabs, Ipswich, MA, USA). This was followed by further purification, native barcode ligation using the PCR Barcoding Expansion (Oxford Nanopore Technologies, Oxford, UK), and adapter ligation with the Adapter Mix II Expansion protocol (Oxford Nanopore Technologies, UK).

The genomic material at each step was quantified using the Qubit dsDNA Quantification Assay Kit (Life Technologies, Carlsbad, CA, USA). Following barcoding, the samples were pooled and loaded into an Oxford Nanopore MinION R9.4.1 flow cell, primed according to the manufacturer’s instructions using the Flow Cell Priming Kit (Oxford Nanopore Technologies, UK). Sequencing was conducted on the ONT MinION Mk1B device using MinKNOW software.

The initial sequencing run generated raw data in FAST5 format, capturing electrical signals. These files were processed for base calling, converting them to FASTQ format, and demultiplexed using Guppy software, developed by ONT [12].

### 2.3. De Novo Genome Assembly and Taxonomic Classification

The resulting FASTQ files were analyzed using the open-source metagenomic cloud-pipeline provided by the Chan-Zuckerberg IDseq platform [13]. This analysis involved a series of steps: initial quality assessment using Fastp software [14], followed by the removal of human host sequences using minimap2 [15]. Subsequently, the remaining reads were de novo-assembled using Flye software, specifically employing the metaFlye parameter tailored for metagenomic analysis [16]. The resulting contigs and unaligned reads were then classified using the DIAMOND software [17] and cross-referenced with the NCBI Nucleotide database (NT). The complete list of detected microorganisms is provided in Supplementary Table S1.

### 2.4. Phylogenetic Analysis

Pathogens with genome coverage exceeding 80% were subjected to genome assembly using the Genome Detective online tool [18]. The resulting consensus genomes were then assessed for quality and completeness using CheckV software [19]. These genomic sequences were aligned to reference genomes retrieved from the NCBI database using MAFFT with default parameters [20]. Manual curation was performed using Aliview [21] to eliminate biological artifacts. Phylogenetic trees were constructed using Maximum Likelihood (ML) estimation performed by IQ-TREE2, employing the general time reversible (GTR) model of nucleotide substitution and supported by 1000 bootstrap replicates, with the command iqtree -s archive.fasta -m GTR+F+R5 -bb 1000. The final visualization of the phylogenetic trees was facilitated using FigTree software. The datasets used to build the phylogenetic trees are in Supplementary Table S2.

## 3. Results

In this study, nine hospitalized patients were diagnosed with acute febrile illness (AFI), while three exhibited symptoms indicative of severe neurological disease (SND). Notably, two AFI patients lacked sufficient epidemiological data, resulting in their exclusion from further analysis. Table 1 summarizes the epidemiological data of the included patients, showing an age distribution ranging from 14 to 61 years. The gender distribution was equal, with 50% female and 50% male participants. All patients were residents of Minas Gerais, predominantly from Belo Horizonte, although some were from nearby cities such as Ouro Preto or Ribeirão das Neves.

Clinically, the hospitalized patients presented a variety of symptoms that necessitated their admission. Those with AFI commonly reported fever, myalgia, arthralgia, and headaches. In contrast, SND cases were primarily associated with symptoms like headaches, seizures, and potential neurosyphilis. A total of 14 samples were analyzed from these 12 patients, with 2 individuals (patients 7 and 11) contributing 2 samples each. The types of samples included five whole blood, two cerebrospinal fluid, five serum, and two RNA Tempus blood samples.

**Figure 2:**
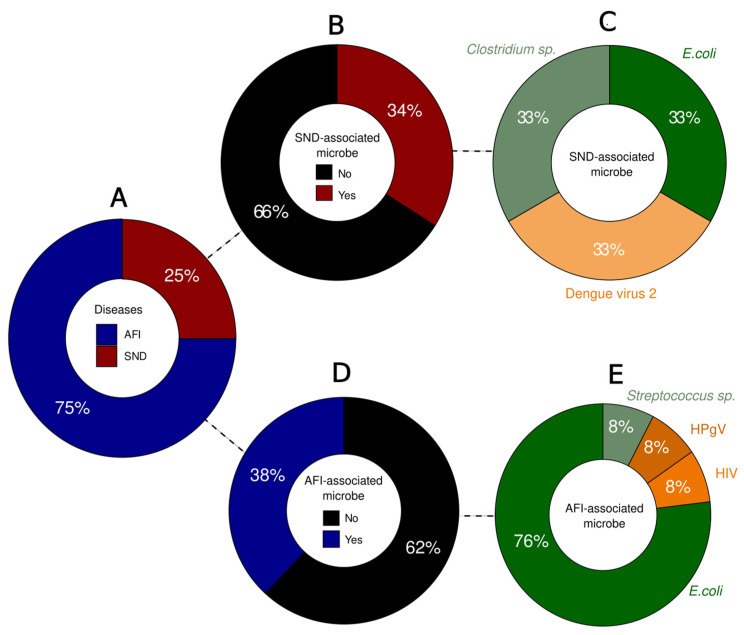
Overview of the proportion of cases per classification and microorganisms identified. (**A**) Percentage of cases per clinical classification. (**B**) Proportion of SND-associated microorganisms in the samples. (**C**) SND-associated microorganisms identified: *Clostridium* sp. (light green), *E. coli* (dark green) and DENV-2 (light orange). (**D**) Proportion of AFI-associated microorganisms in the samples. (**E**) AFI-associated microorganisms identified: *E. coli* (dark green), *Streptococcus* sp. (light green), HIV (orange) and HPgV (dark orange).

Among the patients, 25% were clinically classified as SND cases (n = 3), while 75% were classified as AFI cases (n = 9) (Figure 2A). Among the SND cases, nine microorganisms were identified, with three pathogens (34%) associated with SND: *Escherichia coli* (33%), *Clostridium* sp. (33%), and Dengue virus type 2 (33%) (Figure 2B,C).

Regarding the AFI cases, a total of 21 microorganisms were identified, with 8 (38%) of them being pathogens associated with AFI symptoms (Figure 2D). Bacterial detection was predominant, with *Escherichia coli* (76%) being the most prevalent, followed by *Streptococcus* sp. (8%). Furthermore, viruses were identified in two cases, including Human Immunodeficiency Virus 1 (HIV-1) (8%), and Human pegivirus (HPgV) (8%) (Figure 2E).

Among the bacteria and fungi detected, *Escherichia coli* was identified in all types of collected samples and was associated with a variety of symptoms including fever, myalgia, arthralgia, fatigue, headaches, skin lesions, petechiae, and non-specific signs of neurosyphilis and meningitis. While *E. coli* is often considered part of the normal microbiome, it can also act as a pathogen, particularly when it is the sole pathogen detected or depending on the type of sample in which it is found.

*Clostridium* species were identified in a serum sample from a hospitalized patient with SND presenting severe headache symptoms. Given the range of diseases caused by different species of *Clostridium*, their detection cannot be overlooked. A similar situation occurred with *Streptococcus* species, which were found in a whole blood sample from an AFI patient exhibiting symptoms of fever, myalgia, headaches, and petechiae. In the sequencing control, no microorganisms were detected in the contigs, which highlights the absence of cross-contamination.

**Figure 3:**
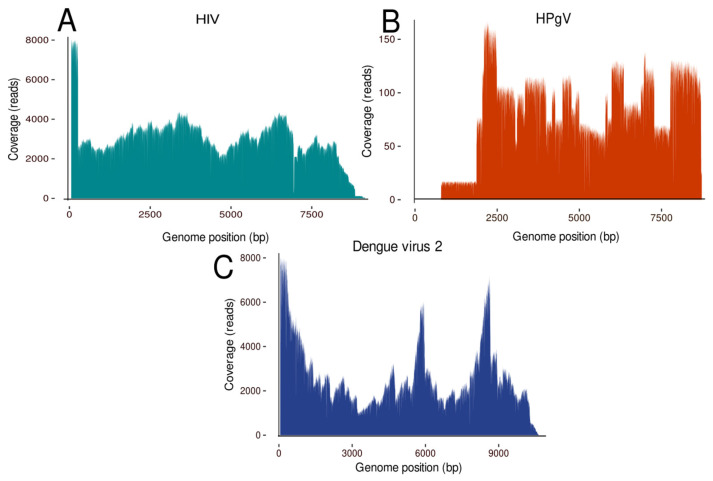
Genome coverage of the viral pathogens identified. (**A**) The genome coverage of the HIV genome, (**B**) the genome coverage of the HPgV genome, and (**C**) the genome coverage of the Dengue virus 2 genome.

The disease-associated bacterial detections had a diverse range of read counting, with some bacterial detections presenting a high number of reads. However, all exhibited very low coverage and depth, which hindered further analysis (Table 2). In contrast, the viruses detected were sequenced with a high number of reads and great quality, allowing the assembly of near-complete genomes of HIV, HPgV, and Dengue virus type 2 (DENV-2) with the current protocol. This enabled the construction of a phylogenetic tree to identify viral lineages associated with these infections (Figure 3).

**Figure 4:**
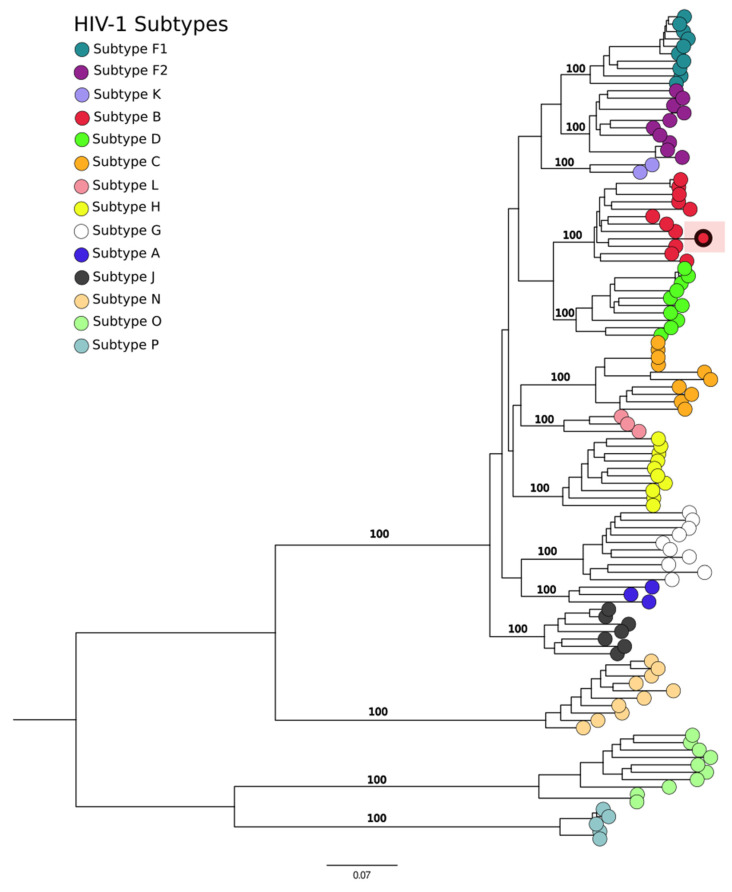
Phylogenetic tree of the HIV genome analysis. The sample from the study is the highlighted sample grouped with subtype B.

The significance of these findings is highlighted by the identification of specific viral subtypes, as demonstrated by the confirmation of HIV in a serum sample from a patient diagnosed with acute febrile illness (AFI). This virus was phylogenetically classified as HIV type 1, subtype B (Figure 4), thereby elucidating the viral subtype present in the patient. This classification enhances our understanding of the pathogenic landscape within the AFI patient cohort.

**Figure 5:**
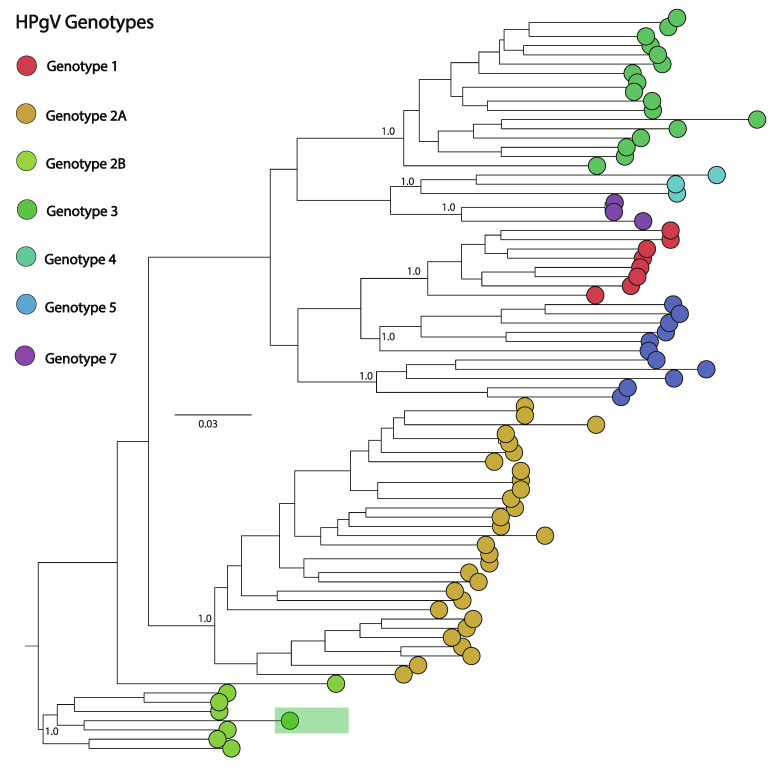
Phylogenetic tree of the HPgV genome analysis. The study’s sample is the one that is highlighted in the human clade.

The HPgV-positive sample was obtained from a whole blood specimen, which also tested positive for *Escherichia coli* and *Streptococcus* species. This sample was associated with symptoms typical of AFI, including fever, myalgia, headaches, and petechiae on the skin. The genome analysis of the sample yielded an 86% complete genome of HPgV. Subsequent phylogenetic analysis classified this virus as belonging to the human strain HPgV-1 (Figure 3C and Figure 5), providing insights into the viral strains affecting this patient population.

**Figure 6:**
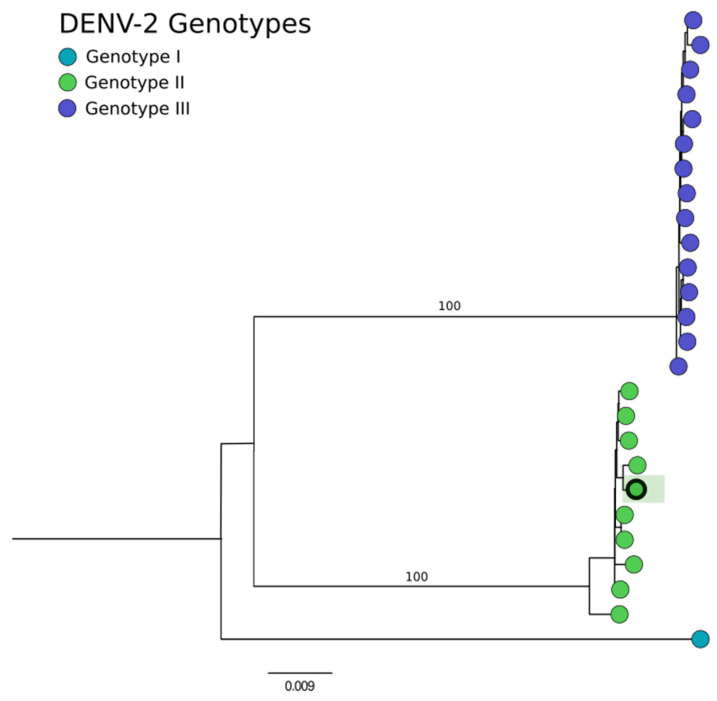
Phylogenetic tree of the DENV2 genome analysis. Comparing the detected genotypes of DENV2 in Brazil, the sample from the study is the highlighted sample that is grouped in genotype II.

From the DENV-2 positive case, a genome was recovered with 99% coverage (Figure 4, which was classified as belonging to genotype II, also known as the cosmopolitan lineage (Figure 6). This virus was detected in a whole blood sample from a patient diagnosed with a severe neurological disorder (SND), presenting with symptoms including headaches, convulsive seizures, and other nonspecific neurological symptoms.

## 4. Discussion

The utilization of metagenomic analysis to elucidate the etiology of AFI and SNDs with unknown origins presents a significant advancement in clinical medicine. The ability to identify a specific etiology can greatly enhance the efficacy of treatments for hospitalized patients. In our research, the application of metagenomic techniques facilitated the identification of diverse microorganisms. This detection capability potentially elucidates the etiological factors underlying the symptoms and resultant hospitalizations of the patients included in the study. Notably, *Escherichia coli* was the most commonly identified pathogen. While *E. coli* is a typical constituent of the human microbiome, under certain conditions, pathogenic strains can lead to various diseases, including acute febrile and neurological disorders [22,23,24,25,26]. The detection of *E. coli* in critical samples such as cerebrospinal fluid, whole blood, and serum necessitates heightened attention due to its potential to trigger systemic inflammatory responses, potentially leading to septicemia [27]. Additionally, our metagenomic analysis recovered nearly complete genomes of several viruses, facilitating further analyses to determine their lineages and genotypes. Such information is crucial for developing targeted treatment strategies and understanding the epidemiological characteristics of these viruses within the population. For example, HIV was identified and classified phylogenetically as HIV type 1, subtype B, which is prevalent both globally and in the Minas Gerais state [28,29]. This subtype was also detected in a patient who had previously reported being HIV-positive but had elected not to pursue treatment in 2020. This case underscores the utility of metagenomic analysis in identifying secondary infections that could explain the patient’s hospitalization for AFI.

The study also highlighted the controversial pathogenicity of HPgV, detected in a sample co-infected with *Streptococcus*. While some studies do not associate HPgV with any specific human diseases, others suggest potential links to conditions such as lymphoma or brain encephalitis [30]. This ambiguity points to the need for further research to clarify the clinical significance of HPgV, particularly when found in conjunction with typical arbovirus-like symptoms such as fever, myalgia, headache, and petechiae. Our findings also identified pathogens associated with SNDs, including *Clostridium* sp. and DENV-2. The presence of the *Clostridium* genus, known for its association with severe neurological symptoms, raises public health concerns, although the specific species could not be determined [31,32]. Moreover, DENV-2 is a well-known neurological pathogen [33,34], which genetically belonged to the genotype II cosmopolitan lineage, recently reported to be spreading in Brazil and also specifically in Minas Gerais State [35,36,37]. This lineage was first detected in the region in April 2022 and has since been prevalent, highlighting the benefits of metagenomic analysis in tracking the spread and evolution of pathogens.

## 5. Conclusions

In conclusion, our metagenomic analysis has yielded significant insights into the pathogens associated with AFI and SND. This comprehensive approach has delineated a spectrum of bacteria, fungi, and viruses, illuminating their potential roles in the pathogenesis of these conditions. The precise identification of these pathogens, along with their genetic profiles and involvement in co-infection scenarios, substantially augments our capacity to formulate timely and specifically targeted therapeutic strategies. Furthermore, this study underscores the critical need for continued research into the pathogenicity of relatively unexplored microbes such as HPgV. The outcomes of this research not only contribute to a deeper understanding of infectious disease dynamics but also enhance the implementation of metagenomic methodologies in clinical diagnostics and public health, thereby laying the groundwork for future exploratory and applied research in this field.

## Figures and Tables

**Table 1:** Epidemiological information from the patients analyzed in this study.

Patient	Gender	Age	City	State	Clinical Classification	Symptoms	Sample
1	Male	53	Belo Horizonte	Minas Gerais	SND	Headaches	Serum
2	Female	38	Belo Horizonte	Minas Gerais	AFI	Fever, myalgia, arthralgia, fatigue, headache, and skin lesions (rash)	Tempus
3	Male	38	Belo Horizonte	Minas Gerais	SND	Headache, convulsive seizure, and neurological symptoms	Whole blood
4	Male	53	Ouro Preto	Minas Gerais	SND	Suggestive of neurosyphilis and bacterial meningitis	Whole blood
5	Female	43	Belo Horizonte	Minas Gerais	AFI	Myalgia, arthralgia, fatigue, and petechiae	Serum
6	Male	34	Belo Horizonte	Minas Gerais	AFI	Fever, myalgia, headache, and petechiae	Whole blood
7	Female	27	Ribeirão das Neves	Minas Gerais	AFI	Fever, cough, myalgia, fatigue, and petechiae	Tempus, serum
8	Female	14	Belo Horizonte	Minas Gerais	AFI	Fever, myalgia, and headache	Whole blood
9	Male	37	Belo Horizonte	Minas Gerais	AFI	Fever	Serum
10	Male	61	Betim	Minas Gerais	AFI	Fever	Serum
11	Female	23	Belo Horizonte	Minas Gerais	AFI	Fever and subacute myeloradiculopathy	Cerebrospinal fluids
12	Female	32	Belo Horizonte	Minas Gerais	AFI	Fever, myalgia, skin lesions, immunosuppression, and acute hepatitis	Whole blood

**Table 2:** Sequencing data from the detected disease-associated microorganisms.

Patient	Sample Type	Pathogen	Reads	Contigs	Coverage	Depth
1	Serum	*Clostridium* sp.	6554	1	0.8%	0.25×
2	Tempus	*E. coli*	63	1	0.1%	0.005×
3	Whole blood	DENV-2	72,078	3	99.8%	1145×
4	Whole blood	*E. coli*	6939	1	0.1%	0.61×
5	Serum	*E. coli*	10,968	1	0.3%	1×
6	Whole blood	HPgV	511	2	86.2%	41×
		*E. coli*	4635	1	0.4%	0.18×
7		*Streptococcus* sp.	1347	1	0.1%	0.17×
	Tempus	*E. coli*	173	1	0.7%	0.012×
	Serum	*E. coli*	9889	1	0.1%	0.95×
8	Whole blood	*E. coli*	5280	1	0.1%	0.41×
9	Serum	HIV-1	52,420	2	92%	1805×
		*E. coli*	13,778	1	0.2%	1.2×
10	Serum	*E. coli*	22,622	1	0.1%	1.6×
11	Cerebrospinal fluid 1	*E. coli*	2084	1	0.1%	0.19×
	Cerebrospinal fluid 2	*E. coli*	1703	1	0.2%	0.11×
12	Whole blood	-	-	-	-	-
13	Negative control	*E. coli*	27	0	-	-

## Data Availability

The data presented in this study are openly available in FigShare at https://figshare.com/account/home#/projects/201114 accessed on 17 June 2024.

## Supplementary Materials

The following supporting information can be downloaded at: https://www.mdpi.com/article/10.3390/genes15070922/s1, Table S1: Complete List of Detected Microorganisms; Table S2: Datasets Used for Phylogenetic Tree Construction.
